# Identification of Age-Related Macular Degeneration Related Genes by Applying Shortest Path Algorithm in Protein-Protein Interaction Network

**DOI:** 10.1155/2013/523415

**Published:** 2013-12-18

**Authors:** Jian Zhang, Min Jiang, Fei Yuan, Kai-Yan Feng, Yu-Dong Cai, Xun Xu, Lei Chen

**Affiliations:** ^1^Department of Ophthalmology, Shanghai First People's Hospital, School of Medicine, Shanghai Jiaotong University, Shanghai 200080, China; ^2^State Key Laboratory of Medical Genomics, Institute of Health Sciences, Shanghai Jiaotong University School of Medicine and Shanghai Institutes for Biological Sciences, Chinese Academy of Sciences, Shanghai 200025, China; ^3^Beijing Genomics Institute, Shenzhen Beishan Industrial Zone, Shenzhen 518083, China; ^4^Institute of Systems Biology, Shanghai University, Shanghai 200444, China; ^5^College of Information Engineering, Shanghai Maritime University, Shanghai 201306, China

## Abstract

This study attempted to find novel age-related macular degeneration (AMD) related genes based on 36 known AMD genes. The well-known shortest path algorithm, Dijkstra's algorithm, was applied to find the shortest path connecting each pair of known AMD related genes in protein-protein interaction (PPI) network. The genes occurring in any shortest path were considered as candidate AMD related genes. As a result, 125 novel AMD genes were predicted. The further analysis based on betweenness and permutation test indicates that there are 10 genes involved in the formation or development of AMD and may be the actual AMD related genes with high probability. We hope that this contribution would promote the study of age-related macular degeneration and discovery of novel effective treatments.

## 1. Introduction

Macular degeneration generally refers to age-related macular degeneration (AMD or ARMD). It is a major cause of blindness and visual impairment in older individuals (>50 years) in Western countries [1, 2]. In China, the disease incidence is 6.04%–11.19% among age 60–69 and rises with the increase of aging population [3]. Its main symptom is central visual damage. Known as one of the hardest eye diseases to treat, the mechanism underlying AMD has not been clear yet.

However, many risk factors have been identified related to AMD occurrence, such as old age, smoking [4], hypertension, oxidative stress [5], and high-energy visible light [6, 7]. Family history and gene mutations are genetic elements for AMD occurrence. The lifetime risk of developing late-stage macular degeneration is 50% for people who have a relative with macular degeneration, versus 12% for people who do not have relatives with macular degeneration [8, 9].

As a complex disease, identification of disease-related genes is prerequisite and persistent. Through previous genetic analysis and experimental validation, numerous genomic loci and a large number of candidate genes have been shown to involve in AMD [10, 11]. However, they just account for part of the AMD pathogenesis. The majority of AMD cases cannot be explained through these known mechanisms. Recently, through meta-analysis of genome-wide association studies (GWAS) for advanced AMD, it was estimated that currently identified loci account for approximately 55% of the heritability of advanced AMD [12]. So, to identify novel genes involved in the formation and development of AMD is necessary and will improve our knowledge about additional pathways and pathological mechanisms of the disease, as well as methods for more effective treatments.

Since the number of human genes is very huge, it is impossible to screen them to discover novel AMD related genes by experiment alone. Computational methods give an alternative way to help scientists pick out genes that are AMD related genes with higher probability than others. On the other hand, computational methods have been successfully used to tackling various problems in many biological areas, such as protein attributions prediction [13–17], drug design [18–22], and analysis of complicated biological network [23]. In this study, we proposed a novel computational method to identify novel AMD related genes. Based on the current known AMD related genes, retrieved from Retina International and some previous published documents, some novel candidate AMD related genes were discovered by applying Dijkstra's algorithm [24] in the protein-protein interaction network. Further GO and KEGG pathway analysis indicates that some candidate genes are involved in the formation and development of AMD. Thus, they may be the actual AMD related genes with high possibility, which should be paid more attention by scientists. It is hopeful that our contribution would help to uncover the mechanism of this disease and discover novel effective treatments.

## 2. Materials and Methods

### 2.1. Known AMD Related Genes

The AMD related genes were collected from the following resources: (1) Sixteen genes are found in Retina International at the website http://www.retina-international.org/files/sci-news/remacdy.htm (recent update from March 24, 2010), with “age-related macular dystrophy” selected as key words. (2) The genes for the complement system proteins factor H (CFH), factor B (CFB), and factor 3 (C3) are strongly associated with a person's risk for developing AMD. HTRA1 (encoding a secreted serine protease), which has implications for the disease, was identified in 2006 [25, 26]. (3) Mutational analysis of ABCR in juvenile macular degeneration (STGD) families revealed a total of 19 different mutations including homozygous mutations in two families with consanguineous parentage. These data indicate that ABCR is the causal gene of STGD [27]. (4) Deletion of the complement factor H-related genes CFHR3 and CFHR1 protects against AMD [28, 29]. (5) The AMD gene consortium identified 19 loci with associations reaching *P* < 5 × 10^−8^, including seven new loci after genome-wide association studies (GWAS) and meta-analysis. The 23 nearby genes were selected [30]. Finally, we obtained 36 known AMD related genes after integration of all resources, which are listed in Table 1.

### 2.2. Protein-Protein Interaction (PPI) Network

The PPI network was constructed according to the information retrieved from the well-known database STRING (Search Tool for the Retrieval of Interacting Genes/Proteins, http://string.embl.de/) (version 9.0) [31], a large database containing direct (physical) and indirect (functional) interactions. In the database, each interaction consists of two proteins and a score quantifying the likelihood that an interaction may occur. For convenience, let *Q*(*p*_1_, *p*_2_) denote the score of the interaction between two proteins *p*_1_ and *p*_2_. The constructed network took proteins as its nodes, and the edge between any two nodes existed if and only if the score of the interaction between the corresponding proteins was greater than zero. To reflect the difference of interactions, each edge with endpoints *v*_1_ and *v*_2_ in the network was labeled an edge weight defined as follows:

(1) $$\begin{array}{c} w\left(v_{1},v_{2}\right)=1000-Q\left(p_{1},p_{2}\right), \end{array}$$

where *p*_1_ and *p*_2_ were corresponding proteins of nodes *v*_1_ and *v*_2_, respectively. Some previous studies have shown that proteins with an edge connecting them in the network, known as interactive proteins, are more likely to share common features than those without edge connecting them [14–16, 32]. Accordingly, it can be deduced from this fact that proteins with small distance in the network may share similar features.

### 2.3. Calculation of Betweenness Using Dijkstra's Algorithm

As described in Section 2.1, 36 genes were collected, which are related to AMD and must have some common features related to AMD. According to Section 2.2, it can be deduced that the proteins in the shortest path connecting any pair of actual AMD related genes may all share some common features related to AMD. To obtain the shortest path, the Dijkstra's algorithm, a well-known shortest path algorithm conceived by Dijkstra in 1956, was employed [24].

For the given node in a network, its betweenness is a value that is related to the number of the shortest paths containing it as an inner node among all shortest paths connecting all pair of nodes [33]. Since betweenness accounts for direct and indirect influences of proteins at distant network [34], it has been used for studying various natural and man-made networks [33, 35–38]. Here, we used the concept of betweenness to identify novel AMD related genes from known 36 genes. Compared to the original concept of betweenness, we only considered the shortest paths, finding by Dijkstra's algorithm in PPI network, which connected two known AMD related genes instead of all shortest paths. The betweenness of a node *v* in this study is defined as the number of shortest paths containing *v* as an inner node among these shortest paths.

### 2.4. Identification of Related Genes Based on Betweenness and Permutation Test

The betweenness of a node/gene in this study means the number of shortest paths that connect all pairs of known AMD related genes/nodes and contain the node/gene as an inner node. Therefore, it is possible that genes/nodes with high betweenness may share more features related to AMD than those with low betweenness. As for proteins with betweenness equal to 0, we considered the likelihood that they are the novel AMD related genes to be zero. Accordingly, we picked out proteins with betweenness greater than 0 and they are termed as shortest path genes.

The betweenness of some nodes/genes may be influenced by the essential structure of the network. For example, the cut-vertex of the network may always receive high betweenness regardless of the distribution of known nodes/genes. To avoid this situation, a permutation test was conducted to further screen the shortest path genes. We randomly selected the same number of nodes/genes as the actual number of AMD related genes from PPI network 500 times and recalculated the shortest paths between these randomly selected genes. The permutation FDR of the shortest path genes was defined as

(2) $$\begin{array}{c} \text{FD}{\text{R}}_{i}=\frac{\text{count}\left(\text{betweennes}{\text{s}}_{\text{random}}>\text{betweennes}{\text{s}}_{\text{actual}}\right)}{500}, \end{array}$$

where betweenness_actual_ was the number of shortest paths among actual AMD related genes across shortest path gene *i*, betweenness_random_ was the number of shortest paths among randomly selected genes across shortest path gene *i*, and count  (betweenness_random_ > betweenness_actual_) was the count of times when betweenness_random_ was greater than betweenness_actual_. According to Jiang et al.'s work [38], smaller permutation FDR of one shortest path gene indicates that it is the actual AMD related gene with high possibility.

### 2.5. KEGG and GO Enrichment Analysis

Functional annotation tool of DAVID [39] was used for KEGG pathway and GO enrichment analysis. The enrichment *P* value was corrected to control family-wide false discovery rate under certain rate (e.g., ≤0.05) with Benjamin multiple testing correction method [40]. All the genes in the human genome were selected as background during the enrichment analysis. There are 13 items in the output of DAVID; their meanings are listed as below:

1. category: DAVID category, that is, KEGG or GO;
2. term: gene set name;
3. count: the number of genes associated with this gene set;
4. percentage: calculated by “gene associated with this gene set”/“total number of query genes;”
5. *P* value: modified Fisher exact *P* value;
6. genes: the list of genes from your query set that are annotated to this gene set;
7. list total: the number of genes in your query list mapped to any gene set in this ontology;
8. pop hits: the number of genes annotated to this gene set on the background list;
9. pop total: the number of genes on the background list mapped to any gene set in this ontology;
10. fold enrichment: the ratio of the proportions on query genes and the background information which are associated with the gene set;
11. Bonferroni: Bonferroni adjusted *P* value;
12. Benjamini: Benjamini adjusted *P* value;
13. FDR: FDR adjusted *P* value.

## 3. Results and Discussion

### 3.1. Candidate Genes Filtered by Betweenness and Permutation Test

For the 36 known AMD related genes, we searched the shortest path connecting any pair of them. After counting the betweenness of inner nodes in these paths, we obtained 168 shortest path genes with betweenness greater than 0, where 4 are known AMD related genes. These 168 genes are available in Supplementary Material I available online at http://dx.doi.org/10.1155/2013/523415. To further screen these genes, the permutation test was conducted and permutation FDRs (refer to (2)) of these genes were calculated, which are also listed in Supplementary Material I. It can be seen that 125 genes were with permutation FDRs less than 0.01, which are considered to be greatly related to AMD.

### 3.2. Results of Functional Annotation Tool

Functional annotation tool of DAVID was used to analyze the 125 genes based on KEGG pathways and GO terms. The GO and KEGG analysis results can be found in Supplementary Materials II and III, respectively. The following sections give the detailed discussion based on these results.

### 3.3. GO Enrichment Analysis

**Figure 1:**
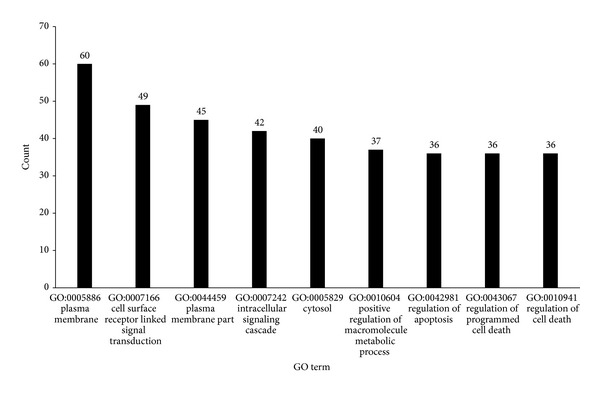
The top 9 GO terms shared by 125 genes. The *X*-axis lists GO's ID and name, while the *Y*-axis represents the number of genes shared the GO term among the 125 genes.

It is observed from Supplementary Material II that 865 GO terms were enriched by the 125 genes. The top 1% of GO terms (totally 9) sorted by “Count” item (refer to Figure 1 for the “Count” item of these GO terms) were investigated and discussed as below.

Among these 9 GO terms, three of them are cellular component (CC) GO terms and six biological process (BP) GO terms. The three CC terms included: GO:0005886: plasma membrane (“count” = 60), GO:0044459: plasma membrane part (“count” = 45), and GO:0005829: cytosol (“count” = 40). Early AMD usually takes place accompanied by accumulation of drusen, a kind of cellular debris, between the retina and the choroid [41]. It was speculated that exosomes produced by cellular lysosome formed the drusen [42]. They carried many extra- or intracellular proteins, lipids, and cellular components wrapped by or included plasma membrane, and gathered other extracellular factors to deposit as a accumulative core [43]. These indicated the role of cytosol parts and plasma membrane in the pathogenesis of AMD in the early stage.

The six BP terms were GO:0007166: cell surface receptor linked signal transduction (“count” = 49), GO:0007242: intracellular signaling cascade (“count” = 42), GO:0010604: positive regulation of macromolecule metabolic process (“count” = 37), GO:0042981: regulation of apoptosis (“count” = 36), GO:043067: regulation of programmed cell death (“count” = 36), and GO:0010941: regulation of cell death (“count” = 36). The first two may represent all the processes of signal transduction. The macromolecule metabolic process was relatively critical in the development of AMD. As mentioned above, metabolism of proteins and other biological macromolecules cannot go without lysosomes. And if that does not work, the exosomes carrying these undigested molecules would induce the formation of drusen or further cause increased local immune responses and promote the AMD. The next three were all related with cell death. It was reported that necrosis can be responsible for the cell loss in the retinal degeneration [44]. Immune responses can protect cells from death and kill them when they were impaired. Advanced AMD in mice model involved death of photoreceptor cells [45]. These genes may not be the causative factors in AMD, but what they have in common would give us a suggestion about how the AMD happens or what problems may occur in the development.

### 3.4. KEGG Pathway Enrichment Analysis

**Figure 2:**
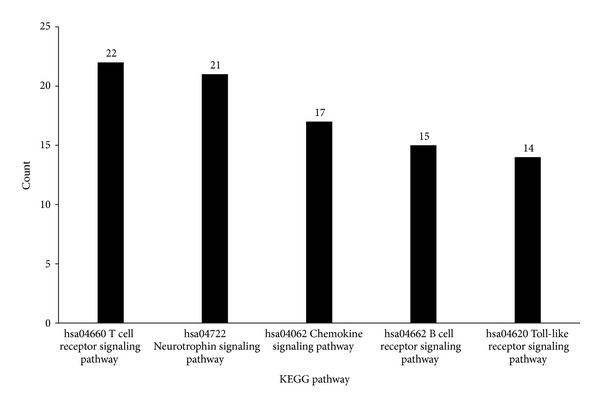
The 5 KEGG pathways shared by 125 genes. The *X*-axis lists pathway's ID and name, while the *Y*-axis represents the number of genes shared the pathway among the 125 genes.

It can be observed from Supplementary Material III that there were 56 pathways which were significantly enriched in by the 125 genes potentially being related with AMD pathogenesis. The KEGG pathways with “count” item at least 14 were investigated. We found that 5 of them are related to AMD.  Figure 2 shows the “count” item of these pathways. In details, T-cell receptor signaling pathway (hsa04660) and neurotrophin signaling pathway (hsa04722) ranked in the top five, covered 22 genes and 21 genes (refer to Figure 2), respectively. Seventeen genes (“percentage” = 13.6%) enriched in the Chemokine signaling pathway (hsa04062), 15 genes (“percentage” = 12%) in the B cell receptor signaling pathway (hsa04662), and 14 genes (“percentage” = 11.2%) in the Toll-like receptor signaling pathway (hsa04620). Although the exact mechanism of AMD is unknown, decades of studies have highlighted the critical role of immune processes in the development, progression, and treatment of AMD [46]. Advanced AMD is of great relevance for the retinal neovascularization and atrophic lesions involving macrophages, lymphocytes, microglia, and mast cells, as well as fibroblasts [47, 48].

Undeniably, all immune responses have dual, opposing roles, normally preventing the host while promoting disease under disorder conditions. The Chemokine signaling pathway is initiated by cytokine-cytokine receptor interactions. It was found that animal models with CC-chemokine ligand 2 (CCL2), CC-chemokine receptor 2 (CCR2), and/or CX3C-chemokine receptor 1 (CX3CR1) deficiency had AMD-like features, including thickening Bruch membrane, deposition of drusen, retinal pigmented epithelium (RPE) hypertrophy, and retinal thinning [49, 50]. T cell receptor signaling pathway and Toll-like receptor signaling pathway are both activated to release immunologic factors, such as IL-1*β*, IL-2, and IL-18. RPE cells can release IL-1*β* in responding to kinds of chemokines which may be dangerous signals and were abundant in AMD [51–53]. While IL- 1*β* and Toll-like receptor 2 (TLR2) ligands can promote neovascularization, activating TLR3 would decrease choroidal neovascularization and promote RPE degeneration [54–56].

B cells do not show a direct role in mediating angiogenesis or tissue damage in AMD pathogenesis, but it cannot be excluded in which there is some relationship between B cell immune and AMD. For example, the Ccl2−/− Cx3cr1−/− mouse model was found having retina-specific autoantibodies [49, 50]. The neurotrophin signaling pathway involves many nerve growth factors (NGFs) and neurotrophins (NT3, NT4). Microenvironment balance of the eye would guarantee the health of eye organisms. It was successful to relieve patients with AMD with NGF eye drop, which suggested the lack of NGF may be a cause of the AMD symptoms and may involve in the disease progress [57]. All the above indicated the relationship of pathways with AMD and may expand the avenues to explore new mechanisms in AMD occurrence and development.

### 3.5. Analysis of 10 Significant AMD Related Genes

There were 12 genes (listed in Table 2) whose betweenness was larger than 100 among the 125 shortest path genes, where 1 gene is the known AMD related gene. Among the rest 11 genes, we found that 10 of them are related to the formation or development of AMD.

Genetic studies had implicated immune responses especially the complement system in AMD pathogenesis and development [43, 58, 59]. The genes for the complement system proteins factor H (CFH), factor B (CFB), and factor 3 (C3) are strongly associated with AMD susceptibility. For instance, the Y402H variant in CFH gene is significantly associated with AMD in Asian populations [60]. C3 can protect the aging retina by complement system [61]. There were two complement component receptors (CR1 and CR2) genes in our result. As c3b/c4b or c3d receptors, the signaling was definitely disturbed if they were dysfunctional. CD19 was a B-lymphocyte antigen. It was reported that the RPE can inhibit the B-cell activation [62], maybe through the B-cell surface antigens, such as cd19.

PSEN1, also known as PS1, was an Alzheimer's disease (AD) gene. The transgenic mice with this gene can express AMD features under some conditions [63], which suggested some internal relationship between the gene and AMD. APP was also an AD gene and would be involved in AMD too. The UBC (ubiquitin C) belonged to the ubiquitin-proteasome system; one terminal hydrolase of which was closely related with photoreceptor cell apoptosis [64]. CBL (also named E3 ubiquitin protein ligase), EGFR, and P53 were both tumor related genes, so their ordinary condition was all necessary for cells growth or organ development, especially the photoreceptor cells' development [65–67]. The Wnt/*β*-Catenin Signaling had been involved in retina development in many studies, and its activities can protect photoreceptors from damage and take part in the retina cell regeneration [68, 69]. Although these genes did not show directly relationship with AMD, their roles indicated some connection with the disease and that would be a cue for further researches to find out more AMD related factors.

## 4. Conclusion

Identification of disease genes is one of the most important and challenging problems in biomedicine and genomics. Since AMD has a high risk factor which results in a loss of vision for elder adults, it is eager to find novel AMD related genes, thereby understanding its mechanism well and discovering effective treatments. This study proposed a computation method to identify AMD related genes by applying the shortest path algorithm in the PPI network. Through the GO and KEGG pathway analysis of the genes identified by our method, they are significantly enriched for GO terms and KEGG pathways related to AMD, which implies they have direct or indirect relationship with the formation or development of AMD. It is hopeful that this contribution would promote the study of this disease.

## Supplementary Material

The description of the Supplementary Material is as follows: "The Supplementary Material contains three files. In details, Supplementary Material I lists 168 shortest path genes identified by our method; Supplementary Material II lists the GO analysis results of 125 genes; Supplementary Material III lists the KEGG analysis results of 125 genes"

## Figures and Tables

**Table 1:** AMD related genes.

Index	Gene name	Index	Gene name
1	ABCR	2	ADAMTS9
3	APOE	4	ARMS2
5	B3GALTL	6	C2
7	C3	8	CETP
9	CFB	10	CFH
11	CFH1	12	CFHR1
13	CFHR3	14	CFI
15	COL10A1	16	COL8A1
17	DDR1	18	ERCC6
19	FBLN5	20	FBLN6
21	FILIP1L	22	HMCN1
23	HTRA1	24	IER3
25	LIPC	26	LOC387715
27	QRX	28	RAD51B
29	RAXL1	30	SLC16A8
31	TGFBR1	32	TIMP3
33	TLR3	34	TLR4
35	TNFRSF10A	36	VEGFA

**Table 2:** Shortest path genes with betweenness greater than 100.

Ensemble ID of genes	Gene name	Betweenness
ENSP00000245907^a^	C3	336
ENSP00000313419	CD19	319
ENSP00000356024	CR2	319
ENSP00000264033	CBL	295
ENSP00000275493	EGFR	195
ENSP00000344456	CTNNB1	172
ENSP00000344818	UBC	158
ENSP00000269305	TP53	114
ENSP00000415941	C4B	111
ENSP00000284981	APP	104
ENSP00000326366	PSEN1	104
ENSP00000356016	CR1	101

^a^It is the known AMD related gene.
