# Supplementary material for: Identification of Age-Related Macular Degeneration Related Genes by Applying Shortest Path Algorithm in Protein-Protein Interaction Network
Source: Biomed Res Int. 2013 Dec 18;2013:523415. doi: 10.1155/2013/523415 (PMC3878555; doi:10.1155/2013/523415)
Supplement: Supplementary file 1 — The description of the Supplementary Material is as follows: "The Supplementary Material contains three files. In details, Supplementary Material I lists 168 shortest path genes identified by our method; Supplementary Material II lists the GO analysis results of 125 genes; Supplementary Material III lists the KEGG analysis results of 125 genes" [file 523415.f1.zip › Supp-I.pdf]

Supplementry Material I. 168 Shortest path genes with betweenness greater than zero and their permutation FDRs

Ensemble ID of genes	Gene name	Betweenness	Permutation FDR
ENSP00000245907[a]	C3	336	0
ENSP00000313419	CD19	319	0
ENSP00000356024	CR2	319	0
ENSP00000264033	CBL	295	0
ENSP00000275493	EGFR	195	0
ENSP00000344456	CTNNB1	172	0
ENSP00000344818	UBC	158	0
ENSP00000269305	TP53	114	0
ENSP00000415941	C4B	111	0
ENSP00000284981	APP	104	0
ENSP00000326366	PSEN1	104	0
ENSP00000356016	CR1	101	0
ENSP00000350941	SRC	98	0
ENSP00000269571	ERBB2	95	0
ENSP00000339007	GRB2	95	0
ENSP00000263253	EP300	91	0
ENSP00000356399	CFH	72	0
ENSP00000252486	APOE	68	0
ENSP00000262160	SMAD2	68	0
ENSP00000264657	STAT3	58	0
ENSP00000360266	JUN	49	0
ENSP00000226574	NFKB1	48	0
ENSP00000401303	SHC1	47	0
ENSP00000211998	VCL	38	0
ENSP00000215631	GADD45B	38	0
ENSP00000221494	SF3A2	38	0
ENSP00000233242	APOB	38	0
ENSP00000236850	APOA1	38	0
ENSP00000243776	CHPF	38	0
ENSP00000245323	EFNB2	38	0
ENSP00000252034	ELN	38	0
ENSP00000261769	CDH1	38	0
ENSP00000262613	SLC9A3R1	38	0
ENSP00000263980	SLC9A1	38	0
ENSP00000267996	TPM1	38	0

ENSP00000268171	FURIN	38	0
ENSP00000275815	EPHA1	38	0
ENSP00000277541	NOTCH1	38	0
ENSP00000281821	EPHA4	38	0
ENSP00000285379	CA2	38	0
ENSP00000305913	COL8A2	38	0
ENSP00000306866	GABARAP	38	0
ENSP00000319248	ZEB1	38	0
ENSP00000322788	MMP1	38	0
ENSP00000333769	BSG	38	0
ENSP00000338934	EZR	38	0
ENSP00000341562	B4GALNT1	38	0
ENSP00000346437	ATG7	38	0
ENSP00000347978	ERCC5	38	0
ENSP00000353542	SPRR2D	38	0
ENSP00000355572	COA6	38	0
ENSP00000357392	EFNA1	38	0
ENSP00000357731	LOR	38	0
ENSP00000358640	SLC16A1	38	0
ENSP00000363970	MAP1LC3A	38	0
ENSP00000374455	SQSTM1	38	0
ENSP00000375986	MAP3K4	38	0
ENSP00000382193	MYBPC3	38	0
ENSP00000309845	HRAS	38	0.004
ENSP00000219070	MMP2	37	0
ENSP00000252444	LDLR	37	0
ENSP00000287727	ZFYVE9	37	0
ENSP00000340944	PTPN11	37	0
ENSP00000357697	S100A2	37	0
ENSP00000360025	GADD45A	37	0
ENSP00000356395	CFHR3	36	0
ENSP00000333919	BTLA	35	0
ENSP00000347948	TNFRSF14	35	0
ENSP00000340858	B2M	34	0
ENSP00000283635	CD8A	34	0.002
ENSP00000227667	APOC3	33	0
ENSP00000261799	PDGFRB	32	0
ENSP00000351273	CASP8	31	0
ENSP00000215829	SNRPD3	30	0

ENSP00000252622	LSM7	30	0
ENSP00000361405	MMP9	30	0
ENSP00000384273	RELA	30	0
ENSP00000398597	EXOSC6	30	0
ENSP00000337825	LCK	29	0
ENSP00000379625	MYD88	29	0
ENSP00000348634	MYH6	28	0
ENSP00000384675	SOS1	28	0
ENSP00000304903	CD2BP2	24	0
ENSP00000356346	PTPRC	24	0
ENSP00000358490	CD2	24	0
ENSP00000285398	ERCC3	23	0
ENSP00000256443	CDK7	22	0
ENSP00000324806	GSK3B	22	0
ENSP00000226730	IL2	21	0
ENSP00000259808	RIPK1	21	0.002
ENSP00000244007	PLCG1	20	0.002
ENSP00000263923	KDR	19	0
ENSP00000353483	MAPK8	19	0
ENSP00000228307	PXN	18	0
ENSP00000338018	HIF1A	18	0
ENSP00000285021	XPC	15	0
ENSP00000011653	CD4	14	0
ENSP00000350708	RAD23B	14	0
ENSP00000357879	PSMD4	14	0
ENSP00000216797	NFKBIA	14	0.004
ENSP00000361626	YBX1	13	0.006
ENSP00000046794	LCP2	11	0
ENSP00000359206	BTRC	11	0.002
ENSP00000221930	TGFB1	11	0.016
ENSP00000300574	CRK	10	0.004
ENSP00000227507	CCND1	10	0.006
ENSP00000347507	MYH7	10	0.012
ENSP00000329623	BCL2	10	0.02
ENSP00000304895	IRS1	9	0.002
ENSP00000368438	PCNA	9	0.006
ENSP00000361021	PTEN	9	0.028
ENSP00000052754	DCN	8	0
ENSP00000206249	ESR1	8	0

ENSP00000229135	IFNG	8	0
ENSP00000400591	SNRPE	8	0
ENSP00000366135	EXOSC10	8	0.004
ENSP00000301838	FADD	7	0.004
ENSP00000280357	IL18	7	0.008
ENSP00000263341	IL1B	7	0.01
ENSP00000263967	PIK3CA	6	0
ENSP00000162749	TNFRSF1A	6	0.006
ENSP00000358622	IKBKG	6	0.01
ENSP00000339151	IKBKB	6	0.03
ENSP00000218388	TIMP1	5	0
ENSP00000341189	PTK2	5	0.02
ENSP00000251849	RAF1	4	0.002
ENSP00000262158	SMAD7	4	0.006
ENSP00000363868	ABCA1	4	0.006
ENSP00000344115	CDH5	4	0.01
ENSP00000414006	LSM2	3	0
ENSP00000254227	NR0B2	3	0.022
ENSP00000274335	PIK3R1	3	0.032
ENSP00000332973	SMAD3	3	0.138
ENSP00000335153	HSP90AA1	3	0.144
ENSP00000329357	SP1	3	0.34
ENSP00000215832	MAPK1	2	0.002
ENSP00000346300	CRKL	2	0.004
ENSP00000338799	IL6ST	2	0.022
ENSP00000344352	ATF3	2	0.034
ENSP00000359424	CHUK	2	0.042
ENSP00000268035	IGF1R	2	0.044
ENSP00000258743	IL6	2	0.066
ENSP00000367207	MYC	2	0.424
ENSP00000222254	PIK3R2	1	0.002
ENSP00000271628	SF3B4	1	0.008
ENSP00000252506	GADD45G	1	0.014
ENSP00000400326	DDX39B	1	0.014
ENSP00000371138	FKBP1A	1	0.016
ENSP00000360683	PTPN1	1	0.02
ENSP00000380227	ITGA4	1	0.02
ENSP00000253727	NR1H2	1	0.022
ENSP00000274376	RASA1	1	0.028

ENSP00000340210	CD59	1	0.028
ENSP00000231509	NR3C1	1	0.038
ENSP00000264972	ZAP70	1	0.044
ENSP00000354558	MTOR	1	0.062
ENSP00000256897	CCNH	1	0.072
ENSP00000309503	YWHAZ	1	0.08
ENSP00000309103	BAD	1	0.108
ENSP00000266970	CDK2	1	0.156
ENSP00000354394	STAT1	1	0.202
ENSP00000371067	JAK2	1	0.222
ENSP00000262367	CREBBP	1	0.25
ENSP00000362649	HDAC1	1	0.278
ENSP00000346839	FN1	1	0.342
ENSP00000350283	BRCA1	1	0.562
ENSP00000352516	DNMT1	1	0.676
ENSP00000270202	AKT1	1	0.776

a: Ensemble IDs in red are known AMD related genes
